# Aldehyde Dehydrogenase 1, a Potential Marker for Cancer Stem Cells in Human Sarcoma

**DOI:** 10.1371/journal.pone.0043664

**Published:** 2012-08-23

**Authors:** Birgit Lohberger, Beate Rinner, Nicole Stuendl, Markus Absenger, Bernadette Liegl-Atzwanger, Sonja M. Walzer, Reinhard Windhager, Andreas Leithner

**Affiliations:** 1 Department of Orthopaedic Surgery, Medical University of Graz, Graz, Austria; 2 Center for Medical Research, Medical University of Graz, Graz, Austria; 3 Institute of Pathology, Medical University of Graz, Graz, Austria; 4 Department of Orthopaedic Surgery, Medical University of Vienna, Vienna, Austria; University of Navarra, Spain

## Abstract

Tumors contain a small population of cancer stem cells (CSC) proposed to be responsible for tumor maintenance and relapse. Aldehyde dehydrogenase 1 (ALDH1) activity has been used as a functional stem cell marker to isolate CSCs in different cancer types. This study used the Aldefluor® assay and fluorescence-activated cell sorting (FACS) analysis to isolate ALDH1^high^ cells from five human sarcoma cell lines and one primary chordoma cell line. ALDH1^high^ cells range from 0.3% (MUG-Chor1) to 4.1% (SW-1353) of gated cells. Immunohistochemical staining, analysis of the clone formation efficiency, and xCELLigence microelectronic sensor technology revealed that ALDH1^high^ cells from all sarcoma cell lines have an increased proliferation rate compared to ALDH1^low^ cells. By investigating of important regulators of stem cell biology, real-time RT-PCR data showed an increased expression of c-Myc, β-catenin, and SOX-2 in the ALDH1^high^ population and a significant higher level of ABCG2. Statistical analysis of data demonstrated that ALDH1^high^ cells of SW-982 and SW-1353 showed higher resistance to commonly used chemotherapeutic agents like doxorubicin, epirubicin, and cisplatin than ALDH1^low^ cells. This study demonstrates that in different sarcoma cell lines, high ALDH1 activity can be used to identify a subpopulation of cells characterized by a significantly higher proliferation rate, increased colony forming, increased expression of ABC transporter genes and stemness markers compared to control cells. In addition, enhanced drug resistance was demonstrated.

## Introduction

The cell population of most tumors is heterogeneous with regard to its proliferation capacity and the ability to initiate tumor formation in immune-deficient mice. A cancer stem cell (CSC) is defined as a cell within a tumour that possesses the capacity to self-renew and to generate the heterogeneous lineages of cancer cells that comprise the tumor [1], [2]. Numerous investigations have provided evidence that CSCs exist in a variety of human tumors such as hematopoietic malignancies, brain tumors, breast cancer, and gastroenterological cancer [3], [4], [5], [6].

Cytosolic aldehyde dehydrogenases (ALDHs) are a group of enzymes involved in oxidizing a wide variety of intracellular aldehydes into their corresponding carboxylic acids [7]. Among theses enzymes, ALDH1 is throught to have an important role in oxidation of alcohol and vitamin A and in cyclophosphamide chemoresistance. Ginestier et al. [8] showed that ALDH1 was a marker of normal and malignant human mammary stem cells and a predictor of poor clinical outcome of breast cancer patients. High ALDH1 activity has been used to define stem cell populations in many cancer types including human multiple myeloma, acute myeloid leukemia [8], pancreatic cancer [9], and breast cancer [10]. Therefore, ALDH1 activity might be usable as a common marker for malignant stem cell populations [11]. Failure of cancer chemotherapy can occur through increased efflux of chemotherapeutic agents, leading to the reduction of intracellular drug levels and consequent drug insensitivity. ABC transporters have the capacity to export many cytotoxic drugs and recent evidence suggests that the cancer stem cell phenotype is associated with high-level expression of the ABCG2 transporter [12], [13], [14].

In this study, we used the Aldefluor® assay and fluorescence-activated cell sorting (FACS) analysis to isolate ALDH1^high^ cells from five human sarcoma cell lines and one recently established chordoma cell line. We analyzed ALDH1^high^ cells *in vitro* for their repopulation capacity, clonogenicity, cell proliferation properties, the expression of stem cell markers and ABC transporters, and their multidrug resistance capacities.

**Table 1:** Primer Sequences used for real-time RT-PCR.

target gene	name	primers	oligonucleotide sequence 5′-3′
ABCG2/BCRP1	breast cancer resistance protein	forward	ACC TGA AGG CAT TTA CTG AA
		reverse	TCT TTC CTT GCA GCT AAG AC
ABCA2	ABCA2	forward	AGA TGG ACA AGA TGA TCG AG
		reverse	GCT TGT ACT TCA GGA TGA GG
ABCB1/MDR1	multidrug resistance protein	forward	GAG GAA GAC ATG ACC AGG TA
		reverse	CTG TCG CAT TAT AGC ATG AA
c-Myc	v-myc myelocytomatosis	forward	GGA ACG AGC TAA AAC GGA GCT
	viral oncogene homolog	reverse	GGC CTT TTC ATT GTT TTC CAA CT
β-catenin	cadherin-associated protein	forward	CCA GCC GAC ACC AAG AAG
	beta 1	reverse	CGA ATC AAT CCA ACA GTA GCC
SOX-2	SRY (sex determining region	forward	CGA GTG GAA ACT TTT GTC GGA
	Y)-box 2	reverse	TGT GCA GCG CTC GCA G
GAPD	glyceraldehyde 3-phosphate	forward	AAGGTCGGAGTCAACGGA
	dehydrogenase	reverse	ACCAGAGTTAAAAGCAGCCCT
hprt-n	hypoxanthine	forward	ATGGGAGGCCATCACATT
	phosphoribosyltransferase	reverse	ATGTAATCCAGCAGGTCAGCAA
ACTB	β-actin	forward	CTGGAACGGTGAAGGTGACA
		reverse	AAGGGACTTCCTGTAACAATGCA

## Materials and Methods

### Cell Culture

All human sarcoma cell lines (SW-684, SW-872, SW-982, SW-1353, and TE-671 were obtained from CLS (Eppelheim, Germany) and cultured in Dulbecco’s-modified Eagle’s medium (DMEM-F12) containing 10% foetal bovine serum (FBS), 1% L-glutamine, 100 units/ml penicillin, 100 µg/ml streptomycin and 0.25 µg amphotericin B. MUG-Chor1 cells were cultured in IMDM/RPMI 1649 (4∶1) (PAA, Pasching, Austria) supplemented with 1% L-glutamine and 1% ITS (PAA). All cell incubation was carried out at 37°C in a humidified atmosphere of 5% CO2 and cultures are periodically checked for mycoplasma. Culture medium and supplements were purchased from GIBCO®, Invitrogen (Darmstadt, Germany).

**Figure 1:**
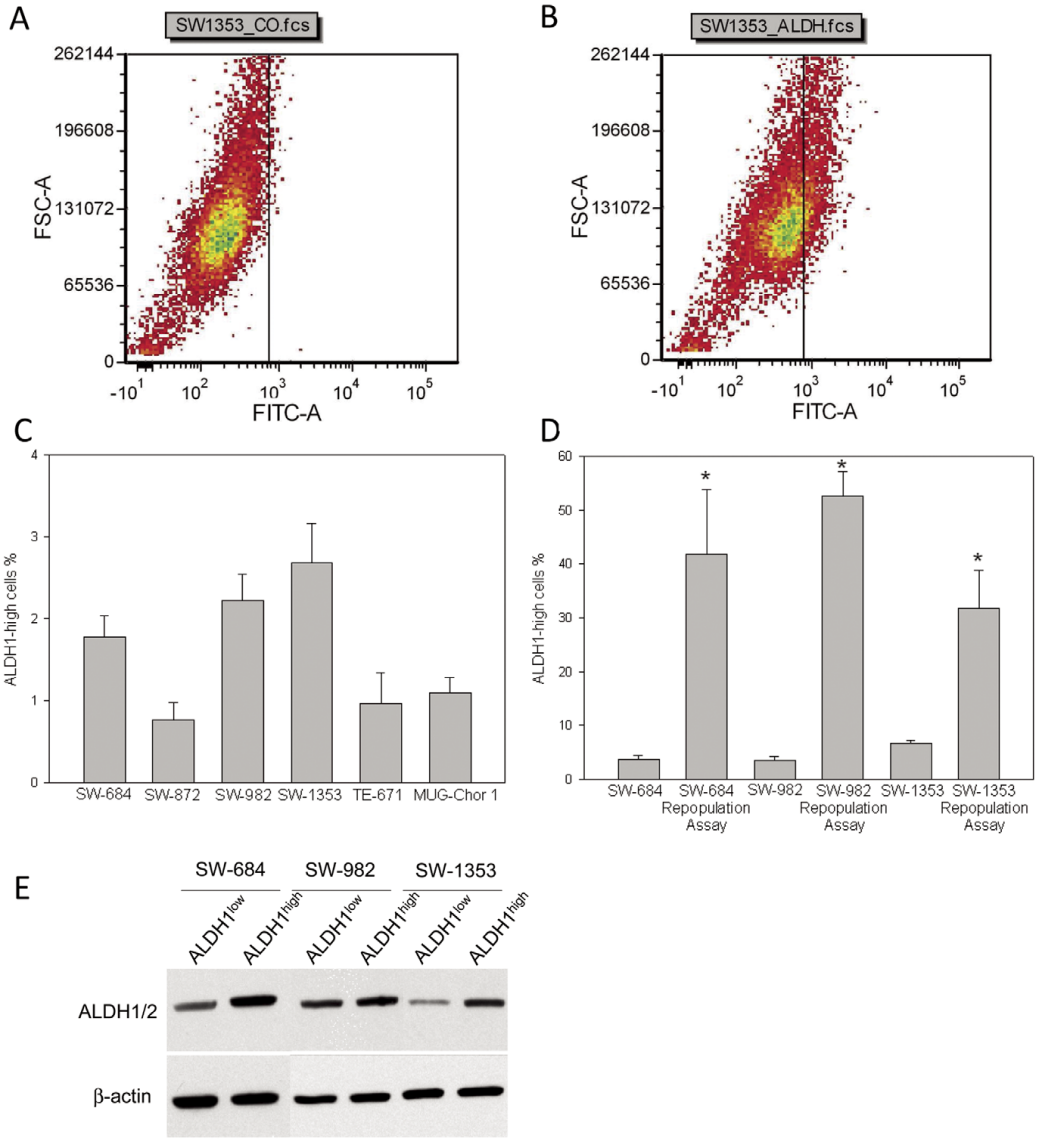
Aldehyde dehydrogenase 1 (ALDH1) expression in sarcoma cell lines using the Aldefluor® assay. Fluorescence versus forward scatter was shown in a density blot from (A) DEAB control cells and (B) ALDH1-expressing cells (called ALDH1^high^). (C) ALDH1 expression in % of gated cells. The highest proportion of ALDH1^high^ cells is represented by SW-684 cells (1.77±0.9%; n = 12), SW-982 cells (2.23±1.0%; n = 11), and SW-1353 cells (2.69±1.3%; n = 8). (D) After two weeks cultured the ALDH1^high^ population generated a significant higher account of ALDH1^high^ cells. (E) The enhanced ALDH activity was also demonstrated by western blot.

**Table 2:** Overview of all results including the corresponding significances.

	SW-684		SW-982		SW-1353	
Method	ALDH1^high^	ALDH1^low^	ALDH1^high^	ALDH1^low^	ALDH1^high^	ALDH1^low^
% ALDH1^high^ cells	1.77±0.9		2.23±1.0		2.69±1.3	
Aldefluor® Assay						
% ALDH1^high^ cells	41.7±18.6		52.5±8.7		31.7±11.1	
Repopulation Assay	*p = 0.0039*		*p = 4.4E-7*		*p = 0.0025*	
expression (%)	67.17%	32.83%	55.34%	44.66%	72.57%	27.43%
ALDH1 western blot						
Ki-67 positivity	0.85±0.17	0.38±0.05	0.59±0.31	0.35±0,27	0.98±0.01	0.18±0.15
Quantitation IHC						
CFE %	4.95±2.59	5.16±2.25	19.0±3.42	15.8±7.66	10.5±2.74	6.5±2.41
colony formation assay					*p = 0.0047*	
c-Myc	1.72±1.44	1.33±1.04	1.52±0.69	0.99±0.40	2.33±1.77	0.82±0.33
relative gene expression						
β-catenin	0.98±0.82	0.87±0.87	0.41±0.06	0.81±0.18	1.16±0.13	0.95±0.37
relative gene expression						
SOX-2	25.2±19.4	9.66±2.77	1.96±1.29	1.02±0.59	4.37±3.83	39.82±28.61
relative gene expression						
ABCG2/BCRP1	2.42±1.56	1.05±0.15	2.28±0.78	1.15±0.29	4.38±0.73	1.76±0.23
relative gene expression	*p = 0.0143*		*p = 0.0047*		*p = 0.0227*	
ABCA2	1.04±0.34	0.94±0.32	3.42±1.37	1.32±0.25	1.88±0.41	0.62±0.17
relative gene expression						
ABCB1/MDR1	1.66±0.61	1.14±0.28	2.25±1.03	1.08±0.27	11.15±2.55	1.52±0.22
relative gene expression					*p = 0.0302*	
doxorubicin IC_50_	1.5 µM	1.4 µM	1.8 µM	0.9 µM	1.1 µM	0.7 µM
drug sensitivity						
epirubicin IC_50_	2.2 µM	2.0 µM	1.3 µM	0.5 µM	1.5 µM	0.7 µM
drug sensitivity						
cisplatin IC_50_	25.7 µM	24.9 µM	23.7 µM	18.8 µM	30.7 µM	26.2 µM
drug sensitivity						

### Aldefluor® Assay and Separation of the ALDH1^+^ Cell Population by FACS Analysis

Aldehyde dehydrogenase (ALDH) enzyme activity in viable cells was determined using a fluorogenic dye based Aldefluor® assay (Stem Cell Technologies, Grenoble, France) according to the manufacturer’s instructions. 1×10^6^/ml cells were suspended in Aldefluor® assay buffer containing ALDH substrate (Bodipy-Aminoacetaldehyde) and incubated for 45 min at 37°C. As a reference control, the cells were suspended in buffer containing Aldefluor® substrate in the presence of diethylaminobenzaldehyde (DEAB), a specific ALDH1 enzyme inhibitor. The brightly fluorescent ALDH1-expressing cells (ALDH1^high^) were detected in the green fluorescence channel (520–540 nm) of FACSAria (BD Biosciences, San Diego, CA) and the data was analyzed using FACS DIVA software (BD Biosciences). To exclude nonviable cells propidium iodide (PI; Sigma Aldrich, Vienna, Austria) was added at a final concentration of 2 µg/ml.

**Figure 2:**
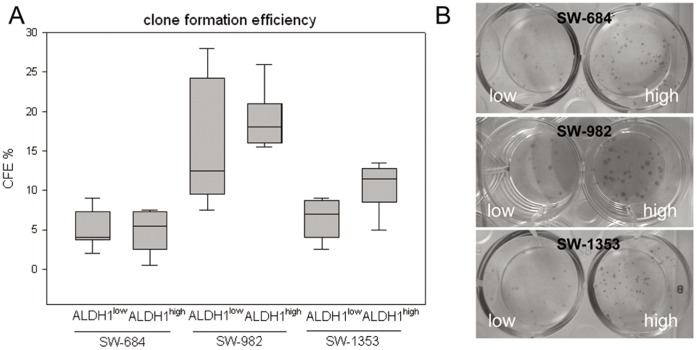
Proliferation analysis of ALDH1^high^ and ALDH1^low^ sarcoma cells. The immunohistochemical analysis using anti-Ki-67 proliferation marker revealed a decreased proliferation level of (A) SW-1353 ALDH1^low^ cells and compared to (B) SW-1353 ALDH1^high^ cells. (C–E) Dynamic proliferation curves for ALDH1^high^ and ALDH1^low^ cells seeded at 10,000 cells per well measured with the xCELLigence system.

### Repopulation Assay

To compare the repopulation ability of sarcoma ALDH1^high^ cells with ALDH1^low^ cells *in vitro*, freshly sorted cells were cultured separately under the same culture condition. After 2 weeks, cells were re-stained with the Aldefluor® assay and reanalyzed via FACSAria (BD Biosciences).

**Figure 3:**
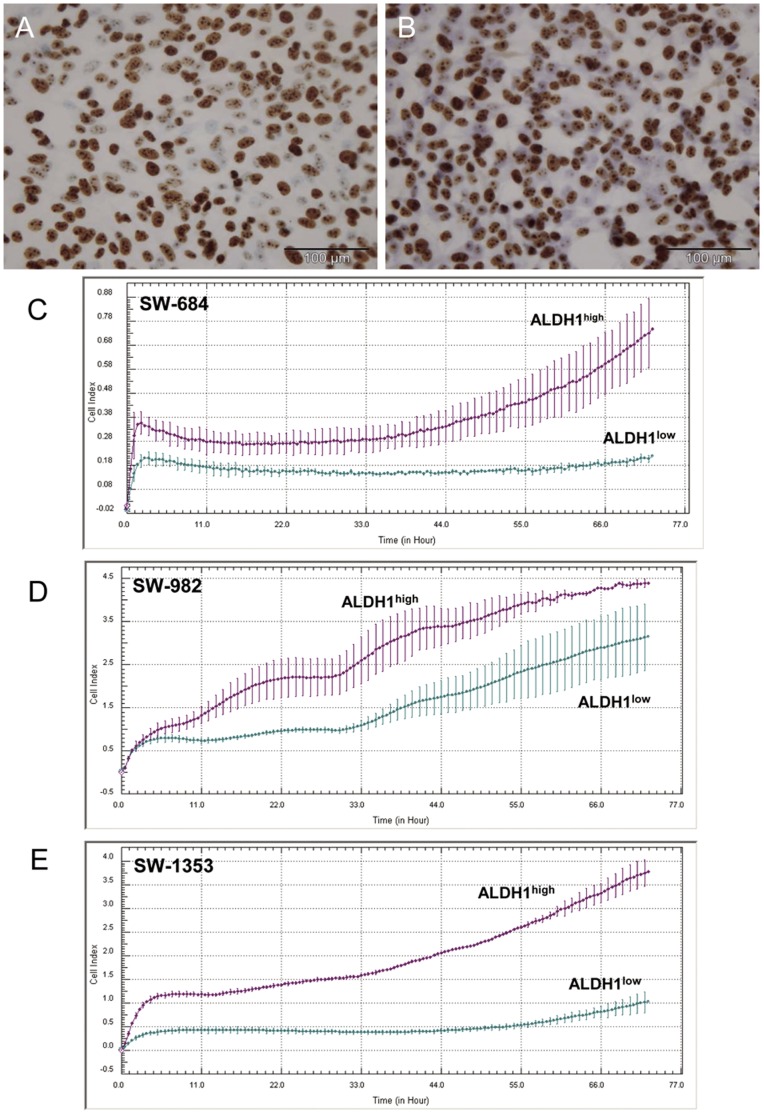
Clonogenic activity of ALDH1^high^ and ALDH1^low^ cells. (A) The quantitation of the clone formation efficiency from SW-684, SW-982, and SW-1353 cells. Data from five independent experiments represent average colony count/well after 14 days. (B) Representative colony forming units from all three cell lines.

### Western Blot Analysis

For total protein analysis, cells were re-suspended in lysis buffer (50 mM Tris-HCL pH 7.4, 150 mM NaCl, 50 mM NaF, 1 mM EDTA, 10% NP-40, 1% Triton-X and protease inhibitors), incubated on ice for 10 min and centrifuged at 15,000 rpm for 15 min. Aliquots of protein extracts (20 µg) were separated on 12% SDS-PAGE and electro-blotted onto 0.45 µm Hybond ECL nitrocellulose membrane (Amersham Biosciences, Little Chalfont, UK). The membrane was blocked with 3% milk blocking buffer for 1 h and then incubated with the primary antibodies for 2 h at room temperature. As the primary antibody, rabbit polyclonal ALDH1/2 antibody (#sc50385; Santa Cruz Biotechnology, Santa Cruz, CA) was used. The major liver isoform ALDH1 localized to cytosolic space, while ALDH2 localized to the mitochondria. The blots were developed using horseradish peroxidase-conjugated secondary antibodies (Dako, Vienna, Austria) at room temperature for 1 h and the SuperSignal® West Pico Chemoluminescent Substrate (Thermo Scientific, Rockford, IL), in accordance with the manufacturers’ protocol.

**Figure 4:**
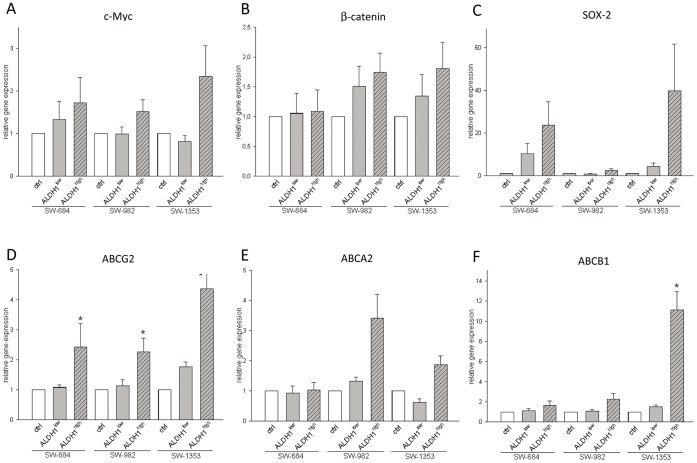
Relative mRNA expression of stemness markers and ABC transporters genes in ALDH1^high^ and ALDH1^low^ cells. The expression level was normalized (ΔC_t_) to the expression of mRNA for GAPDH, ACTB, and hprt-n as an internal control and compared to the corresponding ΔC_t_ (ΔΔC_t_) in controls. The normalized expression levels from (A) c-Myc, (B) β-catenin, and (C) SOX-2 were shown. (D) ABCG2 was more highly expressed in ALDH1^high^ than in ALDH1^low^ cells, whereas the p values for (E) ABCA2 were not significant. (F) ALDH1^high^ SW-1353 cells also showed a significant higher expression of ABCB1.

### Immunohistochemistry

Each 1×10^4^ ALDH^high^ and ALDH^low^ cells were seeded in polystyrene culture slides (BD Biosciences), fixed with 4% formalin/PBS solution, and dehydrated in an ascending series of alcohol. Immunohistochemical (IHC) studies using the streptavidin-biotin peroxidase complex method were carried out employing antibody against the anti- Ki-67 (clone 30-9) rabbit monoclonal primary antibody (Ventana Medical Systems, Tucson, AZ) using the BenchMark Ultra instrument (Ventana Medical Systems). Cells were imaged using an Olympus BX51 microscope with Olympus DP71 microscope digital camera. The stained slides were digitally scanned and positive and negative cells were quantified using the ImageScope software (ImageScope Virtual Slide, version 6.25, Aperio Technol.,Vista, CA). The positivity  = N positive cells/N total cells.

**Figure 5:**
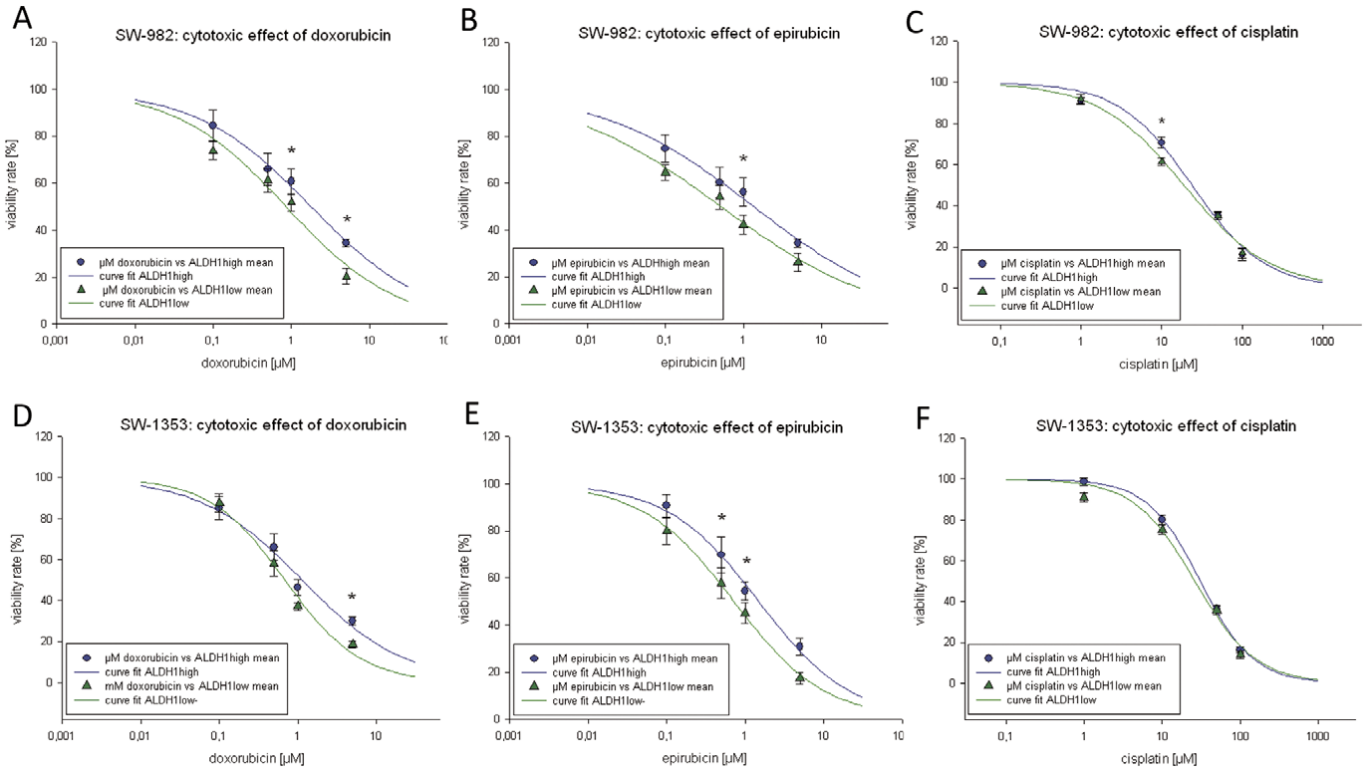
Analysis of the cytotoxic effect of chemotherapeutic agents on ALDH1^high^ and ALDH1^low^ populations sorted from (A–C) SW-982 and (D–F) SW-1353 cells. Both subopulations were treated with 0–5.0 µM doxorubicin, 0–5.0 µM epirubicin, 1–100 µM cisplatin, and measured after 48 h. Mean value ±SD of all measurements was fitted according the Hill equation. Significant differences on the individual concentrations were incorporated in the curves.

### xCELLigence System

The xCELLigence DP device from Roche Diagnostics (Mannheim, Germany) can be used to quantitatively and dynamically monitor cell proliferation in real-time [15]. Respectively 1×10^4^ freshly sorted ALDH1^high^ and ALDH1^low^ cells were seeded in electronic microtiter plates (E-Plate™; Roche Diagnostic) and measured for 72 h with the xCELLigence system according to the instructions in the user’s manual. Application of a low-voltage (less than 20 mV) AC signal leads to the generation of an electric field that interacts with the ionic environment inside the wells of the E-Plates and is differentially modulated by the number of cells in the well, the morphology of the cells, and the strength of cell attachment. Cell density measurements were performed in quadruplicate with a programmed signal detection every 20 min and were normalized to the 6 h time point. Data acquisition and analysis was performed with the RTCA software (version 1.2, Roche Diagnostics).

### Colony Formation Assay

To determine the clone formation efficiency (CFE) of sorted cells *in vitro*, ALDH1^high^, ALDH1^low^ cells and unstained cells (control) were counted and 200 cells per well were seeded in six well plates. Triplicate wells were used for each group. Cells were cultured in DMEM-F12 with supplements for 14 days, fixed in methanol for 10 min and stained with crystal violet (Sigma Aldrich, Hamburg, Germany). The clone’s number which consisted of more than 50 cells was counted. The CFE was calculated according to the formula: (the clone number/the plated cell number)×100.

### Real-Time RT-PCR

Real-time RT-PCR was performed according to MIQE criteria [16] to determine the relative expression of the ABC transporter genes ABCG2/BCRP1, ABCA2, and ABCB1/MDR1 and the stemness markers c-Myc, β-catenin, and SOX-2. Total RNA was isolated with RNeasy Mini Kit (Qiagen, Hilden, Germany) according to the manufacturers’ recommended protocol. DNA was digested with 1 U DNase (Fermentas, St.Leon-Rot, Germany) per µg RNA. 1 µg RNA was reverse transcripted using RevertAid cDNA Synthesis Kit (Fermentas). Real-time PCR reactions were performed in triplicates using the Platinum SYBR Green Super Mix with ROX (Invitrogen) on AB7900HT (Applied Biosystems, Invitrogen). The housekeeping genes glyceraldehyde 3-phosphate dehydrogenase (GAPDH), β-actin (ACTB) and hypoxanthine phosphoribosyltransferase (hprt-n) served as an internal control due to their stable expression in different tissues. Table 1 lists the primers used for real-time PCR. The expression levels were calculated based on the 2^−ΔΔCT^ method [17].

### Drug Sensitivity Assay

Sorted cells were adjusted to a density of 5×10^3^ cells/100 µl and incubated in 96-well microplates. The cells were exposed to various concentrations of chemotherapeutic drugs (doxorubicin hydrochloride, epirubicin hydrochloride, and *cis*-diammineplatinum(II)chloride (cisplatin); Sigma Aldrich) for 48 h. Chemotherapeutic drug sensitivity was determined by the MTS assay (Promega, Mannheim, Germany) following the manufacturers’ instructions in quatruplicates using a photometer (Spektramax; BMG Labtech., Offenburg, Germany) at the wavelength of 490 nm. IC_50_ values were determined from the growth inhibition data.

### Statistical Analysis

The outcome variables were expressed as mean ±SD. Student’s unpaired *t*-test and the exact Wilcoxon’s test was used to evaluate differences between groups with the PASW statistics 18 software (IBM Corporation, Somers, NY). Two-sided *P*-values below 0.05 were considered statistically significant. IC_50_ curves were fitted according the Hill equation (sigmoid, 3 parameters). Graphic data were prepared with SigmaPlot® (Systat Software Inc., San Jose, CA).

## Results

### Sarcoma Cell Lines Display a Distinctive Fraction of ALDH1^high^ Cells

The Aldefluor® assay system has been developed to detect the activity of the ALDH1 isoform. We used this assay followed by FACS analysis to assess the presence and quantity of ALDH1^high^ cell populations in five human sarcoma cell lines and one chordoma cell line [18].

To set a marker for ALDH1^high^ cells DEAB control cells was used to ensure the accuracy of the analysis. The representative SW-1353 cells were treated in the presence of the ALDH1 inhibitor DEAB (Figure 1A) or stained with Aldefluor® reagent, which are defined as ALDH1^high^ cells (Figure 1B). Sorting experiments have been performed a minimum of five times on each cell line. The amount of ALDH1^high^ cells given in average±SD was 1.77±0.9% for the fibrosarcoma cell line SW-684 (n = 12), 0.77±0.4% for the liposarcoma cell line SW-872 (n = 5), 2.23±1.0% for the synovial sarcoma cell line SW-982 (n = 11), and 2.69±1.3% for the chondrosarcoma cell line SW-1353 (n = 8) respectively. The chordoma cell line MUG-Chor 1 showed only a small proportion of 1.11±0.5% ALDH1^high^ cells (n = 9) (Figure 1C). We therefore focused on the three sarcoma cell lines SW-684, SW-982, and SW-1353 in the following experiments. In the repopulation assay the ALDH1^high^ population generated a statistic significant higher account of 41.73±18.5% (*p = 0.0039) ALDH1^high^ cells in the SW-684 cells, 52.5±8.75% (*p = 4.39E-07) in the SW-982 cell line, and 31.7±11.1% (*p = 0.0025) (n = 5) in SW-1353 chondrosarcoma cells (Figure 1D). Additional our observations of enhanced ALDH1 expression could be further be substantiated by western blot analysis (Figure 1E; Table 2).

### ALDH1^ high^ Cells Show Higher Proliferation and Clonogenicity

Using the ImageScope software Ki-67 positive and negative cells were quantified after immunohistochemical staining. ALDH1^high^ cells from all three cell lines have an increased proliferation level compared to ALDH1^low^ cells. Representative staining of SW-982 ALDH1^high^ (Figure 2A) and ALDH1^low^ cells (Figure 2B) are shown and summarized in Table 2 (n = 5). Furthermore, ALDH1^high^ and ALDH1^low^ cells differed significantly in logarithmic growth velocity measured with the xCELLigence-System (Figure 2C−E).

Figure 3A shows the clonogenic activity of ALDH1^high^ and ALDH1^low^ cells. Data from five independent experiments represent average colony count/well after 14 days and all values are listed in Table 2. The clone formation efficiency was significantly higher in SW-1353 ALDH1^high^ cells compared to corresponding ALDH1^low^ cells (*p = 0.0005). For the other two cell lines these effect could also be demonstrated, however in a smaller extent. The higher number of colonies in the SW-684, SW-982, and SW-1353 ALDH^high^ cells is presented (Figure 3B).

### The mRNA Expression of ABCG2, c-Myc, β-catenin, and SOX-2 are Upregulated in ALDH1^ high^ Cells

We investigated whether ALDH1^high^ cells are enriched for expression of genes that have been postulated to play key roles in stem cell biology, such as c-Myc, β-catenin, and SOX-2 [19]. Quantitative RT-PCR showed increased expression of c-Myc in the ALDH1^high^ population, while unsorted control cells (ctrl) and ALDH1^low^ cells had only minimal expression (Figure 4A). Similarly, a slight but not significant increase in the expression of β-catenin, and SOX-2 in the ALDH1^high^ fraction could be observed (n = 6) (Figure 4B–C).

The relative expression of the three major drug transporters ABCG2/BCRP1, ABCA2, and ABCB1/MDR1 was determined by real-time RT-PCR (n = 5). Interestingly the ALDH1^high^ population of all sarcoma cell lines demonstrated, with statistic significance, increased expression levels of ABCG2 compared to control or ALDH1^low^ cells (Figure 4D), whereas the p value for ABCA2 was not significant (Figure 4E). In addition, in ALDH1^high^ SW-1353 cells a statistic significant higher expression of ABCB1 (p = 0.0302) could be observed (Figure 4F). The 2^−ΔΔCT^ values and the corresponding p-values are listed in Table 2.

### ALDH1^high^ Cells Show Enhanced Drug Resistance

The cancer stem cell hypothesis proposes that the discrepancy between treatment response and patient survival noted in most cancer types reflects an inherent resistance of the cancer stem cells to chemotherapy. To investigate possible differences in drug resistance ALDH1^high^ and ALDH1^low^ sorted SW-982 and SW-1353 cells were treated with increasing doses of three commonly used chemotherapeutic agents after a two weeks recovery phase. ALDH1^high^ SW-982 cells treated for 48 h with 1.0 µM (p = 0.016) and 5.0 µM (p = 0.001) doxorubicin were significantly increased compared with ALDH1^low^ cells (Figure 5A). Treatment with 1.0 µM epirubicin (p = 0.045) induced an enhanced drug resistance (Figure 5B). SW-1353 ALDH1^high^ cells showed a similar significant effect after the treatment with 5.0 µM doxorubicin (p = 2.77E-05) (Figure 5D) and 0.5 µM epirubicin (p = 0.039) and 1.0 µM epirubicin (p = 0.021) (Figure 5E). The treatment with cisplatin caused only small differences between ALDH1^high^ and ALDH1^low^ SW-982 and SW-1353 cells (Figure 5C and 5F). In the fibrosarcoma cell line SW-684 no significant differences could be detected. Mean value ±SD of all experiments was fitted according the Hill equation. The corresponding calculated IC_50_ values are listed in Table 2.

## Discussion

Based on the current cancer stem cell (CSC) hypothesis, only a small subpopulation within the heterogeneous tumor population is capable of initiating and re-initiating tumors. The concept of CSCs was based on the observation that when cancer cells of many different types were assayed for their proliferative potential in various assays *in vitro* and *in vivo*, only a minority of cells showed extensive proliferation [20]. CSCs have been identified in a variety of malignancies [21], [22], [23]. One widly accepted method for identifying CSCs is based on the enzymatic activity of aldehyde dehydrogenase 1 (ALDH1), a detoxifying enzyme responsible for the oxidation of intracellular aldehydes [8], [21]. There are different isoforms of ALDH. The Aldefluor® assay system has been developed to detect the activity of the ALDH1 isoform. ALDH1 activity showed to be increased in CSCs and has been used to isolate CSCs in different cancers [24], [25], [26]. Therefore, ALDH1^high^ cells display several features typically seen in CSCs, including the ability for self-renewal, generation of differentiated progeny, and increased expression of stem cell marker genes. The study of CSC biology is predicated on the ability to accurately assess CSC representation within cancer cell populations. As suggested by more recent findings CSC representation may be a function of the cell type of origin, stromal microenvironment, accumulated somatic mutations and stage of malignant progression reached by a tumor [27], [28].

To date, the existence of such a stem-like cell population in human osteosarcoma and Ewing’s sarcoma cell lines has been based on the expression of stem cell marker genes as well as their ability to form spheroids *in vitro* [29], [30], [31]. It has been suggested that identification of the CSC cannot solely rely on side population (SP) sorting using efflux of Hoechst 33342 dye. However the SP phenotype is not presented in all CSCs and there may exist other defensive mechanisms for CSCs to evade drug therapies that cannot be identified by Hoechst dye staining [32]. Therefore, we chose the marker ALDH1. Our results show that all five sarcoma cell lines contained different percentage of ALDH1^high^ cells, with the highest percentage in fibrosarcoma, synovial sarcoma, and chondrosarcoma cell lines. The small ALDH1 expression of the ALDH^low^ cells in the western blot analysis can be explained by the use of the ALDH1/2 primary antibody. The proliferation rate and clonogenicity of SW-684, SW-982, and SW-1353 ALDH1^high^ cells *in vitro* were significantly higher than that of ALDH1^low^ cells, consistent with the characteristics of the high ALDH1 activity phenotype in other cancer cells [33], [34], which may indicate that ALDH1^high^ cells from sarcoma are partially responsible for tumor metastasis and recurrence and should be focused during the cancer therapy. As c-Myc has been recently recognized as an important regulator of stem cell biology, it may serve as a link connecting malignancy and “stemness” [35]. Introduction of c-Myc with other transcription factors (including SOX-2) generates induced pluripotent stem cells from differentiated cells [36]. Wnt/β-catenin signaling plays an important role not only in cancer, but also in cancer stem cells [37]. Our quantitative RT-PCR data showed increased expression of c-Myc, β-catenin, and SOX-2 in the ALDH1^high^ population, while unsorted control cells (ctrl) and ALDH1^low^ cells had only minimal expression.

A proposed mechanism of chemotherapy resistance of cancer stem cells is based on the enhanced expression of ATP-binding cassette (ABC) transport proteins, which are responsible for drug efflux. Higher expression of ABC transport proteins in stem cells compared to non-stem cells results in relative resistance of the stem cells to the toxic effects of chemotherapy drugs [12], [13]. We analysed the mRNA expression of three major drug transporters (ABCG2/BCRP1, ABCA2, ABCB1/MDR1) of ABC transporter family. In the present study, ABCG2 was upregulated in ALDH1^high^ cells from all three sarcoma cell lines. Furthermore, another ABC transporter ABCB1/MDR1 was also found with higher mRNA expression level in SW-1353 ALDH1^high^ cells compared to ALDH1^low^ cells. These genes may be responsible for multi-drug resistance of cancer cells and should be ideal targets for clinical cancer therapy.

Additional, ALDH1^high^ cells showed increased resistance to commonly used chemotherapeutic drugs. ALDH1^high^ cells of SW-982 and SW-1353 showed significantly lower sensitivity to both doxorubicin and epirubicin compared with ALDH1^low^ cells. The cisplatin treatment showed only slight differences. Together, we successfully isolated ALDH1^high^ cells from different sarcoma cell lines using the Aldefluor® assay. ALDH1^high^ cells exhibited *in vitro* a significant higher proliferation rate, increased clone formation efficiency, elevated expression of ABC transporters and stemness marker, as well as increased chemotherapeutic drug resistance compared to ALDH1^low^ cells.

In conclusion, the presence of stem-like cells with increased ALDH1 expression could be one of the possible contributors to the development of drug resistance in sarcomas. Further study will be required to define the sarcoma stem cells and the mechanisms of drug resistance, but ALDH1^high^ population may serve as an *in vitro* model to search for new therapeutic treatment options.

## References

[1] Hajj MA, Clarke MF (2004) Self-renewal and solid tumor stem cells. Oncogene 23: 7274–7282. 15378087 10.1038/sj.onc.1207947

[2] Clarke MF, Dick JE, Dirks PB, Eaves CJ, Jamieson CH, et al (2006) Cancer stem cells - perspectives on current status and future directions: AACR Workshop on Cancer Stem Cells. Cancer Res 66: 9339–9344. 16990346 10.1158/0008-5472.CAN-06-3126

[3] Ponti D, Costa A, Zaffaroni N, Pratesi G, Petrangolini G, et al (2005) Isolation and in vitro propagation of tumorigenic breast cancer cells with stem/progenitor cell properties. Cancer Res 65: 5506–5511. 15994920 10.1158/0008-5472.CAN-05-0626

[4] Scharenberg CW, Harkey MA, Torok-Storb B (2002) The ABCG2 transporter is an efficient Hoechst 33342 efflux pump and is preferentially expressed by immature human hematopoietic progenitors. Blood 99: 507–512. 11781231 10.1182/blood.v99.2.507

[5] Ricci LV, Lombardi DG, Pilozzi E, Biffoni M, Todaro M, et al (2007) Identification and expansion of human colon-cancer-initiating cells. Nature 445: 111–115. 17122771 10.1038/nature05384

[6] Patrawala L, Calhoun T, Broussard RS, Li H, Bhatia B, et al (2006) Highly purified CD44^+^ prostate cancer cells from xenograft human tumors are enriched in tumorigenic and metastatic progenitor cells. Oncogene 25: 1696–1708. 16449977 10.1038/sj.onc.1209327

[7] Vasiliou V, Pappa A, Petersen DR (2000) Role of aldehyde dehydrogenases in endogenous and xenobiotic metabolism. Chem Biol Interact 129: 1–19. 11154732 10.1016/s0009-2797(00)00211-8

[8] Ginestier C, Hur MH, Charafe-Jauffret E, Monvile F, Dutcher J, et al (2007) ALDH1 is a marker of normal and malignant human mammary stem cells and a predictor of poor clinical outcome. Cell Stem Cell 15: 555–567. 10.1016/j.stem.2007.08.014 PMC2423808 18371393

[9] Feldmann G, Dhara S, Fendrich V, Bedja D, Beaty R, et al (2007) Blockade of hedgehog signaling inhibits pancreatic cancer invasion and metastases: a new paradigm for combination therapy in solid cancers. Cancer Res 67: 2187–2196. 17332349 10.1158/0008-5472.CAN-06-3281 PMC3073370

[10] Balicki D (2007) Moving forward in human mammary stem cell biology and breast cancer prognostication using ALDH1. Cell Stem Cell 15: 485–487. 10.1016/j.stem.2007.10.015 18938743

[11] Moreb JS (2008) Aldehyde Dehydrogenase as a Marker for Stem Cells. Curr Stem Cell Res Ther 3(4): 237–246. 19075754 10.2174/157488808786734006

[12] An Y, Ongkeko WM (2009) ABCG2: the key to chemoresistance in cancer stem cells? Expert Opin Drug Metab Toxicol 5(12): 1529–1542. 19708828 10.1517/17425250903228834

[13] Ding XW, Wu JH, Jiang CP (2010) ABCG2: A potential marker of stem cells and novel target in stem cell and cancer therapy. Life Sci 86(17–18): 631–637. 20159023 10.1016/j.lfs.2010.02.012

[14] Gangemi R, Paleari L, Orengo AM, Cesario A, Chessa L, et al (2009) Cancer stem cells: a new paradigm for understanding tumor growth and progression and drug resistance. Curr Med Chem 16(14): 1688–1703. 19442140 10.2174/092986709788186147

[15] Xing JZ, Zhu L, Jackson JA, Gabos S, Sun XJ, et al (2005) Dynamic monitoring of cytotoxicity on microelectronicsensors. Chem Res Toxicol 18: 154–161. 15720119 10.1021/tx049721s

[16] Bustin SA, Benes V, Garson JA, Hellemans J, Huggett J, et al (2009) The MIQE guidelines: minimum information for publication of quantitative real-time PCR experiments. Clin Chem 55(4): 611–622. 19246619 10.1373/clinchem.2008.112797

[17] Vandesompele J, De Preter K, Pattyn F, Poppe B, Van Roy N, et al (2002) Accurate normalization of real-time quantitative RT-PCR data by geometric averaging of multiple internal control genes. Genome Biology 3(7): RESEARCH0034. 12184808 10.1186/gb-2002-3-7-research0034 PMC126239

[18] Rinner B, Froehlich EV, Buerger K, Knausz H, Lohberger B, et al (2012) Establishment and detailed functional and molecular genetic characterisation of a novel sacral chordoma cell line, MUG-Chor1. Int J Oncol 40(2): 443–451. 22002331 10.3892/ijo.2011.1235

[19] Santagata S, Ligon KL, Hornick JL (2007) Embryonic stem cell transcription factor signatures in the diagnosis of primary and metastatic germ cell tumors. Am J Surg Pathol 31: 836–845. 17527070 10.1097/PAS.0b013e31802e708a

[20] Reya T, Morrison SJ, Clarke MF, Weissman IL (2001) Stem cells, cancer, and cancer stem cells. Nature 414: 105–111. 11689955 10.1038/35102167

[21] Li C, Heidt DG, Dalerba P, Burant CF, Zhang L, et al (2007) Identification of pancreatic cancer stem cells. Cancer Res 67: 1030–1037. 17283135 10.1158/0008-5472.CAN-06-2030

[22] O’Brien CA, Pollett A, Gallinger S, Dick JE (2007) A human colon cancer cell capable of initiating tumour growth in immunodeficient mice. Nature 445: 106–110. 17122772 10.1038/nature05372

[23] Schatton T, Murphy GF, Frank NY, Yamaura K, Waaga-Gasser AM, et al (2008) Identification of cells initiating human melanomas. Nature 451: 345–349. 18202660 10.1038/nature06489 PMC3660705

[24] Deng S, Yang X, Lassus H, Liang S, Kaur S, et al (2010) Distinct expression levels and patterns of stem cell marker, aldehyde dehydrogenase isoform 1 (ALDH1), in human epithelial cancers. PLoS One 5(4): e10277. 20422001 10.1371/journal.pone.0010277 PMC2858084

[25] Kim MP, Fleming JB, Wang H, Abbruzzese JL, Choi W, et al (2011) ALDH activity selectively defines an enhanced tumor-initiating cell population relative to CD133 expression in human pancreatic adenocarcinoma. PLoS One 6(6): e20636. 21695188 10.1371/journal.pone.0020636 PMC3113804

[26] Li T, Su Y, Mei Y, Leng Q, Leng B, et al (2010) ALDH1A1 is a marker for malignant prostate stem cells and predictor of prostate cancer patients’ outcome. Lab Invest 90(2): 234–244. 20010854 10.1038/labinvest.2009.127 PMC3552330

[27] Kelly PN, Dakic A, Adam JM, Nutt SL, Strasser A (2007) Tumor growth need not be driven by rare cancer stem cells. Science 317(5836): 337. 17641192 10.1126/science.1142596

[28] Quintana E, Shackleton M, Sabel MS, Fullen DR, Johnson TM, et al (2008) Efficient tumour formation by single human melanoma cells. Nature 456(7222): 593–598. 19052619 10.1038/nature07567 PMC2597380

[29] Awad O, Yustein JT, Shah P, Gul N, Katuri V, et al (2010) High ALDH activity identifies chemotherapy-resistent Ewing’s sarcoma stem cells that retain sensitivity to EWS-FLI1 inhibition. PLoS One 5(11): e13943. 21085683 10.1371/journal.pone.0013943 PMC2978678

[30] Wang L, Park P, Zhang H, La Marca F, Lin CY (2011) Prospective identification of tumorigenic osteosarcoma cancer stem cells in OS99–1 cells based on high aldehyde dehydrogenase activity. Int J Cancer 128(2): 294–303. 20309879 10.1002/ijc.25331

[31] Yang M, Zhang R, Yan M, Ye Z, Liang W, et al (2010) Detection and characterization of side population in Ewing’s sarcoma SK-ES-1 cells in vitro. Biochem Biophys Res Commun 391(1): 1062–1066. 20004177 10.1016/j.bbrc.2009.12.020

[32] Ma S, Chan KW, Hu L, Lee TK, Wo JY, et al (2007) Identification and characterization of tumorigenic liver cancer stem/progenitor cells. Gastroenterology 132: 2542–2556. 17570225 10.1053/j.gastro.2007.04.025

[33] Croker AK, Allan AL (2011) Inhibition of aldehyde dehydrogenase (ALDH) activity reduces chemotherapy and radiation resistance of stem-like ALDHhiCD44+ human breast cancer cells. Breast Cancer Res Treat DOI 10.1007/s10549-011-1692-y. 10.1007/s10549-011-1692-y 21818590

[34] Hellsten R, Johansson M, Dahlman A, Sterner O, Bjartell A (2011) Galiellalactone Inhibits Stem Cell-Like ALDH-Positive Prostate Cancer Cells. PLoS One 6(7): e22118. 21779382 10.1371/journal.pone.0022118 PMC3133629

[35] Murphy MJ, Wilson A, Trumpp A (2005) More than just proliferation: Myc function in stem cells. Trends Cell Biol 15: 128–137. 15752976 10.1016/j.tcb.2005.01.008

[36] Takahashi K, Yamanaka S (2006) Induction of pluripotent stem cells from mouse embryonic and adult fibroblast cultures by defined factors. Cell 126: 663–676. 16904174 10.1016/j.cell.2006.07.024

[37] Teng Y, Wang X, Wang Y, Ma D (2010) Wnt/beta-catenin signaling regulates cancer stem cells in lung cancer A549 cells. Biochem Biophys Res Commun 392(3): 373–379. 20074550 10.1016/j.bbrc.2010.01.028

